# Draft Genome Sequence of the Caffeine-Degrading Methylotroph *Methylorubrum populi* Pinkel

**DOI:** 10.1128/MRA.01300-19

**Published:** 2019-11-21

**Authors:** Rebecca E. Parales, Gaurav Sharma, Xiangsheng Zhang, Gabriel A. Subuyuj, Jordan T. Langner, Matthew E. Wright, Jayna L. Ditty, Scott C. Dawson

**Affiliations:** a Department of Microbiology and Molecular Genetics, College of Biological Sciences, University of California, Davis, Davis, California, USA; b Jiangsu Provincial Key Laboratory of Coastal Wetland Bioresources and Environmental Protection, Yancheng Teachers University, Yancheng, China; c Department of Biology, University of St. Thomas, St. Paul, Minnesota, USA; University of Rochester School of Medicine and Dentistry

## Abstract

A pink-pigmented facultative methylotroph, Methylorubrum populi Pinkel, was isolated from compost by selective enrichment with caffeine (3,5,7-trimethylxanthine) as the sole carbon, nitrogen, and energy source. We report here its high-quality draft genome sequence, assembled in 35 contigs totaling 5,630,907 bp. We identified 5,681 protein-coding sequences, including those putatively involved in caffeine degradation.

## ANNOUNCEMENT

Caffeine (3,5,7-trimethylxanthine) is a natural purine alkaloid synthesized by many plant species. Most of the >35 caffeine-degrading bacterial isolates that have been reported to date are members of the genus Pseudomonas (1). We report here the isolation and draft genome sequence of a facultative methylotroph, Methylorubrum populi Pinkel, which can utilize caffeine as a sole carbon, nitrogen, and energy source, and predict that it uses the *N*-demethylation pathway to convert caffeine to xanthine.

Strain Pinkel was obtained from compost that contained coffee grounds, following enrichment in a variation of BG-11 minimal medium (2) lacking citrate and sodium nitrate, with 0.006 g/liter ferric citrate substituted for ferric ammonium citrate and containing 5 mM caffeine as the sole carbon and nitrogen source. A pink-pigmented pure culture was obtained after multiple passes on plates of the same medium. The strain was identified as Methylorubrum populi (3, 4) based on 16S rRNA gene amplification with universal 16S primers followed by sequence homology analysis. As expected for a Methylorubrum isolate, the strain was a rod-shaped facultative methylotroph capable of growing on methanol as sole carbon source (3, 5). It grew on 5 mM caffeine as the sole source of carbon, nitrogen, and energy with a doubling time of approximately 6.5 h.

Genomic DNA was purified using an ArchivePure DNA kit (5 Prime, Inc., Gaithersburg, MD). The genomic DNA (100 ng) was used to create a paired-end library using the Ovation Ultralow protocol (NuGen, Redwood, CA) by Illumina. Later, the library was sequenced using the Illumina MiSeq platform, producing 1,058,337 paired-end reads (length, 300 bp). Raw reads were quality trimmed with a minimum Phred quality score of 30 from both ends using Trimmomatic v0.35 (6) with default parameters, generating 973,280 (91.96% of total) sequencing reads of good quality. *De novo* assembly and scaffolding were performed using A5-miseq (version 20150522) (7), followed by the removal of duplicate contigs and contigs containing only repeat regions. Finally, contigs were cross-checked for any putative contamination using a Kraken-based taxonomic sequence classification system (8). The final assembly consists of 35 contigs (>500 bp) with a total size of 5,630,907 bp, an *N*_50_ value of 589,043 bp, a G+C content of 69.2%, and ∼40× coverage. The smallest and the largest contigs were 753 bp and 1,232,378 bp, respectively. Subsequently, the genome annotation was carried out with Rapid Annotations using Subsystems Technology (RAST) (9), which predicted a total of 5,885 coding sequences, including 5,681 protein coding sequences, 52 tRNA genes, 2 rRNAs (16S and 23S), and 150 repeat regions. Out of 5,681 proteins, 1,414 (∼25%) of the genes were assigned to SEED subsystems, whereas 4,267 (∼75%) were predicted to have an unknown (nonhypothetical) function. Subsystems signifying the survival of the isolate in aromatic compound-contaminated soil included membrane transport (90 genes), stress response (62 genes), metabolism of aromatic compounds (29 genes), and motility and chemotaxis (93 genes). As expected for a *Methylorubrum* species, the genome contained genes for carotenoid biosynthesis, flagellar motility and chemotaxis, and methanol utilization. In addition, genes for the *N*-demethylation pathway for caffeine degradation were also identified.

### Data availability.

This whole-genome shotgun project has been deposited at DDBJ/ENA/GenBank under the accession number WEKV00000000. The version described in this paper is the first version, WEKV01000000. The data are under BioProject number PRJNA514782 and BioSample number SAMN10160078. The reads are available under SRA accession number SRR8435744.
